# Molecular imaging of MMP activity discriminates unstable from stable plaque phenotypes in shear-stress induced murine atherosclerosis

**DOI:** 10.1371/journal.pone.0204305

**Published:** 2018-10-10

**Authors:** Robert Seifert, Michael T. Kuhlmann, Sarah Eligehausen, Friedemann Kiefer, Sven Hermann, Michael Schäfers

**Affiliations:** 1 European Institute for Molecular Imaging (EIMI), University of Münster, Münster, Germany; 2 Department of Nuclear Medicine, University Hospital Münster, Münster, Germany; 3 DFG EXC 1003 Cluster of Excellence ‘Cells in Motion’, University of Münster, Münster, Germany; 4 Max Planck Institute for Molecular Biomedicine, Münster, Germany; Monash University, AUSTRALIA

## Abstract

**Purpose:**

As atherosclerotic plaque ruptures are the primary cause of ischaemic events, their preventive identification by imaging remains a clinical challenge. Matrix metalloproteinases (MMP) are involved in plaque progression and destabilisation and are therefore promising targets to characterize rupture-prone unstable plaques. This study aims at evaluating MMP imaging to discriminate unstable from stable plaque phenotypes.

**Methods:**

ApoE deficient mice (ApoE^-/-^) on a high cholesterol diet underwent implantation of a tapered cuff around the right common carotid artery (CCA) inducing a highly inflamed atherosclerotic plaque upstream (US) and a more stable plaque phenotype downstream (DS) of the cuff. 8 weeks after surgery, the MMP inhibitor-based photoprobe Cy5.5-AF443 was administered i.v. 3h prior to *in situ* and *ex vivo* fluorescence reflectance imaging of the CCAs. Thereafter, CCAs were analysed regarding plaque size, presence of macrophages, and MMP-2 and MMP-9 concentrations by immunohistochemistry and ELISA.

**Results:**

We found a significantly higher uptake of Cy5.5-AF443 in US as compared to DS plaques *in situ* (1.29 vs. 1.06 plaque-to-background ratio; p<0.001), which was confirmed by *ex vivo* measurements. Immunohistochemistry revealed a higher presence of macrophages, MMP-2 and MMP-9 in US compared to DS plaques. Accordingly, MMP-2 concentrations were significantly higher in US plaques (47.2±7.6 vs. 29.6±4.6 ng/mg; p<0.05).

**Conclusions:**

In the ApoE^-/-^ cuff model MMP-2 and MMP-9 activities are significantly higher in upstream low shear stress-induced unstable atherosclerotic plaques as compared to downstream more stable plaque phenotypes. MMP inhibitor-based fluorescence molecular imaging allows visualization of these differences in shear stress-induced atherosclerosis.

## Introduction

Cardiovascular diseases are common in populations worldwide and mostly originate from atherosclerosis, a chronic inflammation of the arterial vessel wall [1]. This vascular inflammation, the product of various molecular and cellular pathoprocesses, develops over years to decades leading to the formation of a complex atherosclerotic lesion in the vessel wall—the atherosclerotic plaque. The plaque consists of accumulated lipids, inflammatory cells and fibrous tissue, thereby thickening the arterial wall and potentially obstructing the arterial lumen. The plaque potentially limits perfusion of organs such as the heart or the brain. Life-threatening clinical complications of atherosclerosis impend when stable plaques transform into unstable or vulnerable phenotypes, characterized by fragile fibrous caps and a high degree of inflammation. These might suddenly rupture, triggering an immediate occlusion of the plaque-carrying artery with hypoperfusion and hypoxia of the flow-dependent organs, such as the heart or the brain. Therefore, an urgent clinical need exists for novel, non-invasive vascular imaging techniques to discriminate high-risk, unstable plaques from stable ones. These imaging modalities should be able to describe the broad variety of known or emerging inflammatory processes and mechanisms that contribute to plaque vulnerability.

Here we aim at specifically image the activity of matrix metalloproteinases (MMPs) to discriminate stable from unstable plaques–a novel approach in the later described human like atherosclerosis model. This approach is based on findings, that in human carotid plaques MMPs, primarily MMP-9 and MMP-2, are overexpressed and activated in rather vulnerable and symptomatic plaque states as analysed by immunohistochemistry of endatherectomy samples [2] [3]. Interestingly, MMP activity was associated with a thin fibrous cap and a large lipid core phenotype of carotid plaques, making MMP activity a promising molecular target for molecular imaging.

To study the interrelation of shear stress and atherosclerotic plaque phenotypes, Cheng et al. [4] developed a conical tapered cuff model that is implanted around a carotid artery in ApoE^-/-^ mice on a high-cholestrol diet. The surgical constriction primarily alters the blood flow and thereby hemodynamic forces acting on the endothelium resulting in three distinct conditions: low shear stress upstream (US), high shear stress inside and oscillatory shear stress downstream (DS) the tapered cuff [5]. The US region is known to show unstable plaque formation with a rather vulnerable phenotype whereas the DS plaque is typically smaller and exhibits more stable features upon histology [6].

We used this model in the study, as it nicely reflects the clinical scenario of a high-degree carotid artery stenosis and seems ideal to also evaluate new tracers targeting molecular or cellular mechanisms of plaque progression. In a first approach, we aimed at evaluating molecular imaging by [^18^F]FDG-PET (2-deoxy-2-[^18^F]fluoro-D-glucose positron emission tomography) to distinguish inflammation in the advanced US plaque from that in the DS plaque [5].

However, the [^18^F]FDG approach is limited by targeting a rather unspecific molecular process (glucose uptake and phosphorylation), which might also be present in brown fat surrounding the cuff area, and hypoxia-induced [^18^F]FDG uptake inside atherosclerotic plaques [7]. By contrast, here we aim at a highly specific molecular imaging approach by visualising the MMP activity in US and DS plaques in the ApoE^-/-^ cuff model. For this purpose, we make use of a Cy5.5-labelled broad spectrum MMP-inhibitor photoprobe that has been previously developed by our group and shown to bind active MMP molecules [8].

Simultaneous imaging of MMP activity in low as well as high shear stress plaques has not been studied yet, but is obligatory to assess MMPs’ clinical value for the detection of vulnerable atherosclerosis. Thus, this study aims at investigating whether MMP activity could be used as an imaging biomarker to differentiate between stable and vulnerable plaques.

## Materials and methods

### Tapered cuff model and mice

A total of 32 ApoE^-/-^ mice (Imaging: n = 22, of which 6 were used for histomorphometry and 5 for immunohistochemistry; ELISA analysis: n = 15) with a mean age of 18 weeks at start of a Western type diet (see below). Mice were either directly purchased from Charles River Laboratories (Germany) or taken from the own ApoE^-/-^ mice breeding colony and kept in groups of up to 4 animals in macrolon cages with filter cap and provided with enrichment material, stored at constant temperature (22 ± 2°C) and relative humidity (46 ± 4%) in a rodent flow cabinet. Animals had free access to water and food pellets, at first standard mouse chow, which was changed at the beginning of the experiment to a 12-weeks high fat high cholesterol diet (Altromin GmbH, Germany; 21% fat (15% cacoa butter), 0.25% cholesterol, 19.5% casein).

After 4 weeks on diet, a tapered cuff was implanted around the right carotid artery (CCA), whose conical inner lumen constricts the vessel diameter and thereby modifies shear stress in a defined manner [5] [4]. For a detailed description of the implantation procedure see our video and text tutorial [9]. For tracer administration and image acquisition mice were immobilized by isoflurane anaesthesia (2% isoflurane, 0.5 l O_2_/min). All surgeries were performed under combined anaesthesia consisting of isoflurane inhalation (2% isoflurane, 0.5 l O_2_/min) and intraperitoneally injected narcotics (0.04 mg Fentanyl / 4 mg Midazolam / kg bodyweight) for effective pain treatment during the intervention. In order to treat any post-surgical pain carprofen (5 mg/kg bodyweight) was administered s.c. at the end of the surgery and again, if necessary, the day after. To detect any health problems during the 12 weeks of high fat diet the animals were observed on a daily basis. All animal experiments were performed in accordance with the legal requirements of the European Community (Directive 2010/63/EU) and the corresponding German Animal Welfare Law (TierSchG, TierSchVersV) and were approved by the local authorizing agency (State Office for Nature, Environment and Consumer Protection North Rhine-Westphalia; permit no. 84–02.04.2013.A046).

### In vivo and ex vivo fluorescence imaging

**Fig 1:**
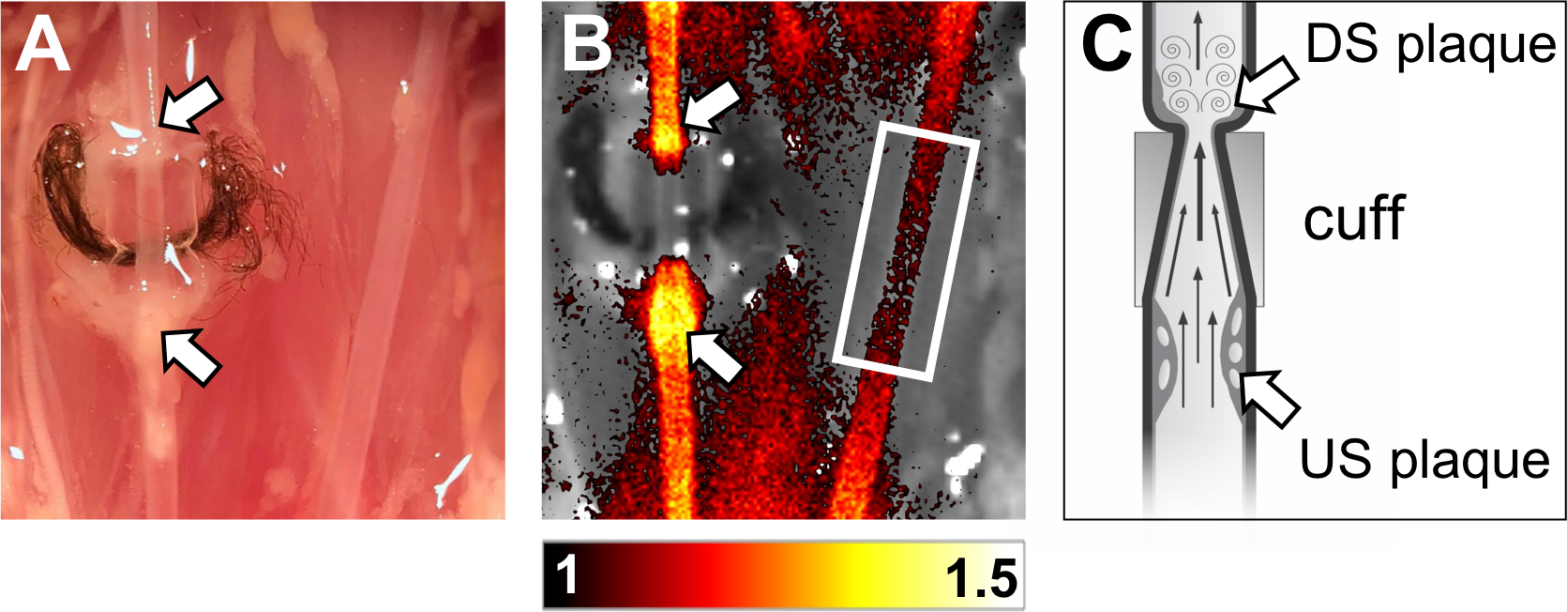
Representative fluorescence reflectance image of carotid artery. Example of preparation situs as well as *in situ* FRI 3 h post injection of Cy5.5-AF443. (A) Situs of left and right common carotid artery. Arrows mark the large upstream (US) plaque formation and the minor atherosclerotic lesion downstream (DS) of the implanted cuff. (B) FRI image of identical situs overlaid to a white light image *in situ*, showing higher signal intensity in the US region. Plaque to background ratio (P/B) is calculated by normalization of FRI to left carotid artery (depicted by white rectangle). For the fluorescence image, plaque to background ratios (P/B) are used as image units. (C) Schematic drawing of the right CCA’s longitudinal section with cuff-induced plaque formation US and DS. Modified from [9].

Animals (n = 22) were kept on diet for another 8 weeks before imaging. Three hours before imaging, 2 nmol Cy5.5-AF443 in 0.1 ml NaCl solution were administered intravenously. For MMP-2 and MMP-9, IC_50_ concentrations of the unlabelled photoprobe are 27 (nM) and 138 (nM) [8]. To assess unspecific tracer uptake, we used the identical but non-targeted dye Cy5.5-glycin as a control in five animals. Please see the S1 Fig for details. Immediately before imaging, mice were sacrificed under deep isoflurane anaesthesia (5%) by whole body perfusion with 0.9% NaCl solution (“bleeding”) followed by perfusion with 4% PFA solution for tissue fixation. Chest and neck were dissected to expose the aortic arch, both CCAs and the descending aorta. Fluorescent reflectance images and white light photographs were acquired to assess the *in situ* tracer uptake. Then both CCA were explanted and re-imaged by FRI to capture the *ex vivo* tracer uptake without background signal from surrounding tissues. Fluorescence reflective imaging (FRI) was carried out using a IVIS Spectrum Imaging System and Living Image Software 4.0, Caliper Life Sciences, Hopkinton, MA, USA (Fig 1). Data is presented as arbitrary units (AU, [p/sec(cm2/sr*10^9^]) or as background normalized ratio (P/B, mean fluorescence intensity of left CCA served as background).

### Image analysis

Images were analysed by a semi-automatic, custom-made MATLAB application (The MathWorks, Inc., Natick, MA, USA). A central vessel path (centreline) was set in the US and DS region, serving as origin for an automated ROI definition. In these ROIs, mean and maximum fluorescence intensities were measured and expressed as arbitrary units (AU, [p/sec(cm2/sr×10^9^]). Measurements in the US and DS region were additionally normalized to background signals (mean fluorescence intensity of left CCA ROI) and expressed as plaque to background (P/B) ratios.

### Histology

After acquiring *ex vivo* images, each right CCA was completely cut into series of 5 μm-thick cross-sectional slices. For each animal, slices representing the DS, the US as well as the cuff region were H&E stained (n = 6 animals) to calculate the area of the vessel lumen and the inner and outer tunica media. To assess the plaque size, the luminal area was subtracted from the area enclosed by the inner layer of the tunica media. Consequently, the so calculated histomorphometrical parameters are named: a) vessel size, b) plaque size and c) lumen size for the remainder of this paper. All image measurements were performed using Fiji software package [10].

To gain complementary histological information, slices adjacent to the H&E stained ones were selected for immunohistological analysis (n = 5 animals): a) MMP-2 DAB staining (primary Antibody: Abcam plc, Cat. # ab 37150, Cambridge, UK) and b) MMP-9 fluorescence staining (primary antibody Abcam plc, Cat. # ab38898, Cambridge, UK), both to correlate the tracer results, c) Mac-3 DAB staining (primary antibody: BD Pharmingen, Cat. # 550292, San Diego, CA, USA) performed as a double staining with d) MRP14 Fast Red staining (primary antibody against MRP14 provided by T. Vogl, Institute for Immunology, University Hospital Münster) to locate macrophages and to assess the overall inflammatory activity in the tissue.

Immunohistologically processed slices were scanned using a Nikon Eclipse Ni-E upright microscope (Nikon GmbH, Düsseldorf, Germany). The resulting photographs were evaluated semi-quantitatively by a consensus reading of four histology experts in 4 grades (0 = no staining signal or no plaque, 1 = single positive cells in plaque, 2 = moderate signal in multiple locations inside plaque, 3 = bold, confluent signal), because an automated threshold staining-quantification was not feasible due to artefacts caused by the plastic cuff. To condense the results, a summed group score was calculated from the consensus reading results of all animals with respect to staining (MMP-2/-9, MRP14 or Mac-3) and region (US, cuff or DS).

### ELISA

ApoE^-/-^ 8 weeks after cuff implantation (n = 15) were sacrificed by whole body perfusion with NaCl solution (see above). The tapered cuff was removed to store DS as well as US regions and the left CCA at -70°C. Tissue samples were homogenized using Omni beads (Omni International, Inc, Kennesaw, GA, USA, Cat. # 19-628-3) and solution buffer (50 mM TRIS HCl, 0.1% Triton X, PBS dilution). Protein concentration was determined by Bradford assays (BIO RAD, Hercules, CA, USA, Cat. # 500–0006). MMP-2 (Quickzyme Biosciences, Leiden, The Netherlands, Cat. # QZBMMP2M) and MMP-9 (Quickzyme Biosciences, Leiden, The Netherlands, Cat. # QZBMMP9M) ELISAs were performed in accordance to manuals. Total MMP concentration was measured using p-aminophenylmercuric acetate (APMA) solution (0.5 mM) as MMP activator. Two animals were excluded in the MMP-2 ELISA and one animal in the MMP-9 ELISA due to a lack of internal consistency. MMP Concentrations were normalized to individual protein levels [ng (MMP) / mg (protein)].

### Statistical analysis

Results are shown as mean +/- standard error of the mean (SEM). Significant differences were assessed using either paired two-sided t-tests or two-sided Wilcoxon testing. Wilcoxon tests were performed for the following analyses: *in situ* imaging P/B, US vs. control; *ex vivo* imaging, US vs DS; *ex vivo* imaging, DS vs control; MMP9 ELISA analysis, US vs control. The Shapiro–Wilk test was used to assess data normality. As US, DS and control sections stem from an individual mouse, a paired test design was used to account for inter-individual differences that could influence test results. Significance tests were performed using Sigmaplot software (Systat Software, Inc., San Jose, CA, USA) and accepted if p < 0.05. Regression analysis testing and plots were performed using MATLAB (The MathWorks, Inc., Natick, MA, USA).

## Results

As expected and previously described, ApoE^-/-^ mice developed the characteristic shear stress-induced plaque phenotypes upstream (“unstable” = large, macrophage-rich, highly inflamed) and downstream (“stable” = smaller, less macrophages, less inflammation) of the cuff 8 weeks after implantation of the tapered cuff. Fig 1A shows a typical *in situ* situation with the macroscopically visible US plaque forming just before the marked cuff. Following injection of the MMP photoprobe Cy5.5-AF443 plaques showed an increased uptake 3h p.i. *in situ* and *ex vivo*, however, with a clear difference in uptake between the upstream and the downstream plaques. An example of FRI of the carotid arteries is given in Fig 1A and 1B). It exemplifies the characteristic distribution of MMP signals, with a stronger signal in the US plaque as compared to the DS plaque, whereas the control CCA only showed a background signal.

### *In situ* fluorescence reflectance imaging

**Fig 2:**
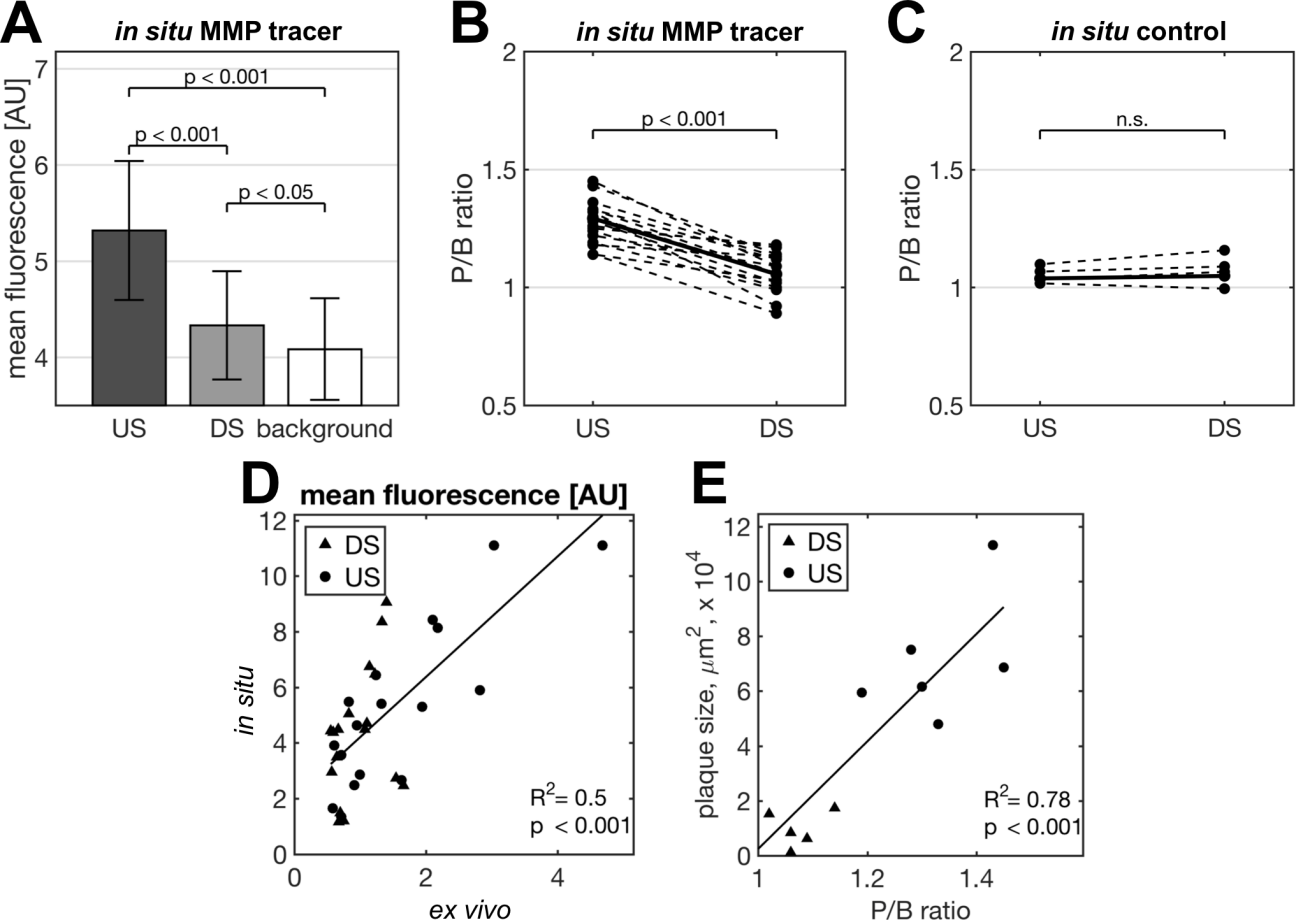
MMP tracer quantification results. Quantification of fluorescence intensity 3 h post injection of Cy5.5-AF443 (n = 17) or the non-targeted control dye Cy5.5-glycin (n = 5). Absolute *in situ* MMP tracer uptake [AU] (A). P/B (plaque to background) ratios of the MMP tracer (B) and of the non-targeted control dye (C). MMP tracer correlation of *in situ* to *ex vivo* findings (D) as well as of P/B ratios to plaque size (E) (n = 6). For panel A, B and C, paired t-tests were performed.

In the 17 ApoE^-/-^ cuff mice studied by FRI the mean fluorescence signal *in situ* was significantly higher in the US as compared to the DS plaque (US 5.3±0.72 AU vs. DS 4.3±0.56 AU; p<0.001; Fig 2A). As compared to the background signal in the contralateral left CCA (4.1±0.52 AU) both the US and DS plaques showed a significantly enhanced mean fluorescence signal (US p<0.001; DS p<0.05; Fig 2A). As expected, additional peak fluorescence signal analyses consolidated and reinforced these findings (US 6.4±0.78 AU vs. DS 4.8±0.62 AU; p<0.001, data not shown). Accordingly, plaque to background (P/B) ratios were higher for US as compared to DS plaques (1.29±0.03 vs. 1.06±0.02; p<0.001; Fig 2B). To investigate potential unspecific tracer uptake, we used the non-targeted labelling dye Cy5.5-glycin alone with no specific affinity to MMP molecules. Using this control dye, there was no significant difference between US and DS plaque to background ratios (1.04±0.02 vs. 1.05±0.04; p>0.05). Please see Fig 2C and S1 Fig for details.

### *Ex vivo* fluorescence reflectance imaging

Since the fluorescent signals observed in planar FRI *in situ* are potentially “contaminated” by unspecific uptake in tissues surrounding the carotid arteries additional FRI measurements of explanted right and left CCAs (with tissues surrounding the arteries removed) *ex vivo* were performed to further validate the *in situ* findings. Again, the mean fluorescence intensity in US plaques was higher than in DS plaques and in the control left CCA (US 1.6±0.27 AU vs. DS 0.97±0.9 AU vs. left CCA 0.81±0.08 AU; US vs DS p<0.01; DS vs left CCA p<0.001). Fig 2D shows a good correlation of *in situ* and *ex vivo* findings. *Ex vivo* P/B ratios were higher for US than for DS plaques 1.85±0.15 vs. 1.23±0.07; p<0.01).

### Vessel size, plaque size and luminal narrowing

**Fig 3:**
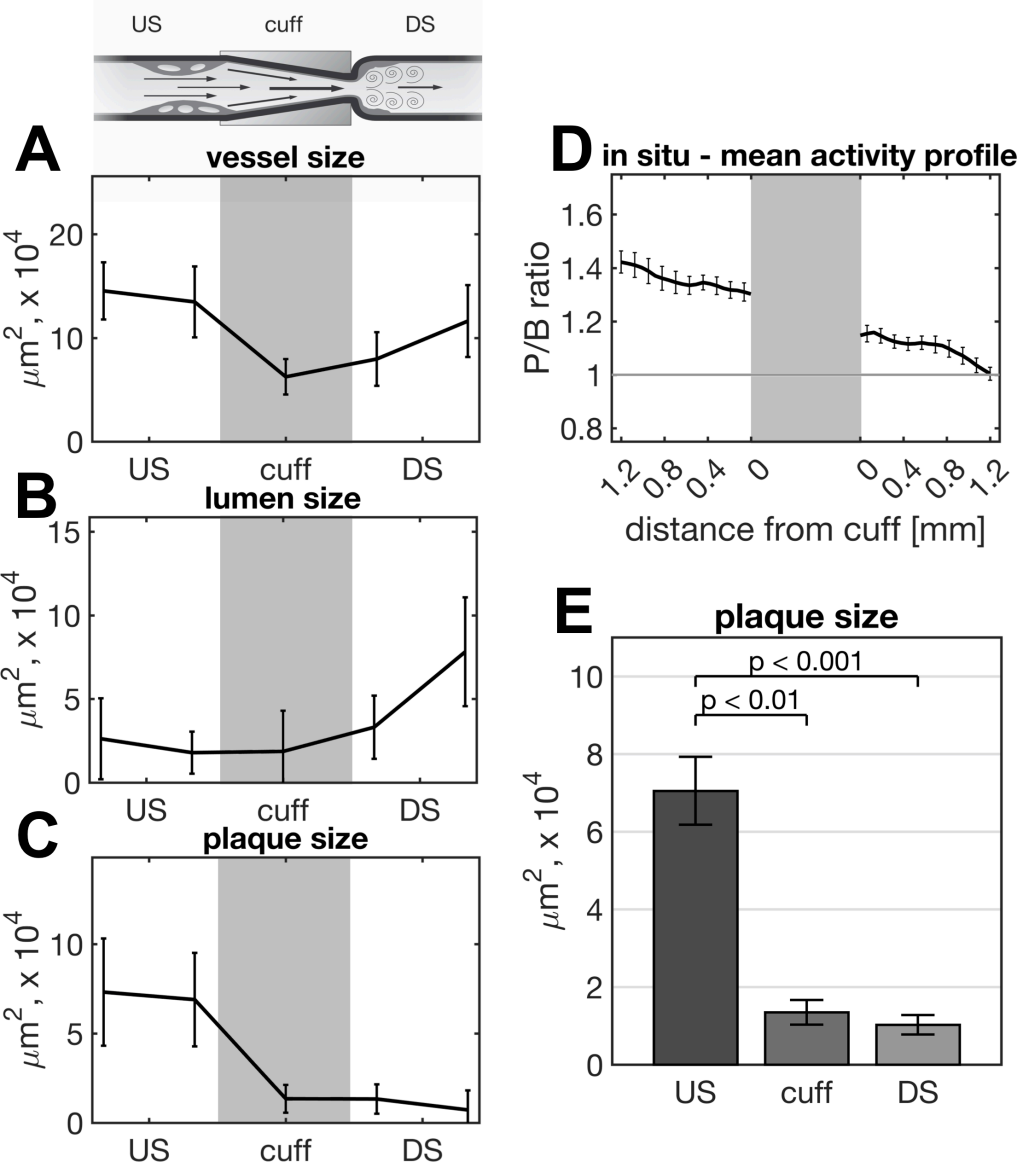
Histomorphometry and imaging profile of the carotid artery. Schematic drawing of the right CCA’s longitudinal section with cuff-induced plaque formation upstream (US) and downstream (DS). Modified from [9]. Mean profiles of the vessel size (A), lumen size (B) and plaque size (C) of the left CCA are shown highlighting the narrowed lumen US due to plaque formation. Mean *in situ* profile of the P/B (plaque-to-background) ratio from FRI images 3 h post injection of Cy5.5-AF443 alongside the right CCA (D). Average US, cuff and DS plaque size showing the significant larger plaque size US compared to DS and the cuff region (E).

To correlate the FRI signals in US and DS plaques with the corresponding histological plaque phenotypes we harvested carotid artery tissues from a total of 5 mice immediately after FRI imaging and performed a detailed histology in these samples. Interestingly, the vessel size upstream of the cuff was not different to that of the downstream portion of the right CCA indicating no significant outward remodelling (vessel size US 14.0±0.87 10^4^ 𝜇m^2^ vs. DS 9.8±1.0 10^4^ 𝜇m^2^; average values; p = ns). However, the US lumen size (2.2±0.55 10^4^ 𝜇m^2^; average value) was significantly smaller than the DS lumen size (5.6±1 10^4^ 𝜇m^2^; average value) due to a larger plaque size of US plaques (7.1±0.78 10^4^ 𝜇m^2^; average value) compared to DS plaques (1.0±0.28 10^4^ 𝜇m^2^; average value; p<0.001), as displayed in Fig 3E. When analysing the FRI signal versus the plaque size, we found a strong correlation between these parameters (*in situ* P/B ratios vs. histological plaque size R^2^ = 0.78, p<0.001, Fig 2E). In addition to the region-of-interest based approach we have also assessed profiles along the right CCA for the histological parameters vessel size, lumen size and plaque size as well as for the FRI signal (Fig 3A–3D). These support the above findings of an equal vessel size up- and downstream of the cuff but a significant stenosis and large plaque volume upstream the cuff which is correlated to an increased FRI signal following injection of the MMP photoprobe.

### Confirmatory histological staining

**Fig 4:**
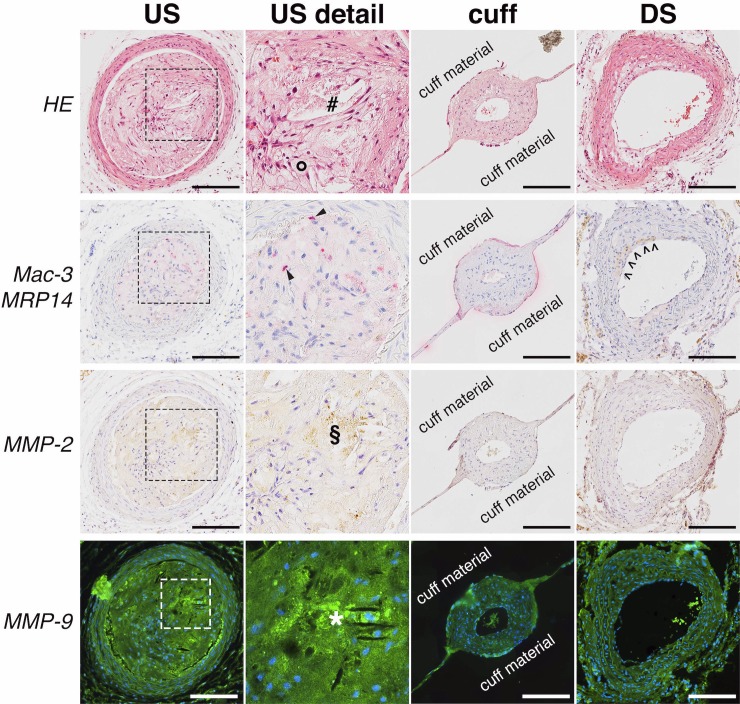
Representative histology of carotid artery. Typical adjacent carotid cross-sections taken straight upstream (US), straight downstream (DS) and within the cuff 8 weeks after implantation, stained for HE, Mac-3/MRP14, MMP-2 and MMP-9. HE staining (first row) shows extensive plaque formation, large foam cells (circle) and a narrowed vessel lumen (number sign) in contrast to relatively mild plaque formation in the DS region. Inside the cuff no plaque formation is visible. Magnifications of the same plaque region from different US sections (dashed boxes) demonstrate red stained MRP14-positive cells (arrow heads) close to an area positive for MMP-2 (section sign) and MMP-9 (asterisk sign). In the MRP14/Mac-3 stained DS section open arrow heads line a stripe of plaque deposition positive for Mac-3 (brownish stain). (Scale bar = 150 𝜇m).

To study the specificity of our imaging approach we correlated individual FRI signals with findings in immunohistochemistry of mice following MMP imaging. To assess the molecular target of our imaging probe, we stained sections of US and DS plaques for MMP-2 and MMP-9 in addition to staining neighbouring slices for macrophages (Mac-3) and for the phagocyte activity marker MRP14 to characterize cellular and molecular inflammatory activity. Fig 4, S2 and S3 Figs illustrate histological features which are characteristic for findings in the larger cohort of ApoE^-/-^ mice studied and are confirming the results of the original work [4] [11].

**Fig 5:**
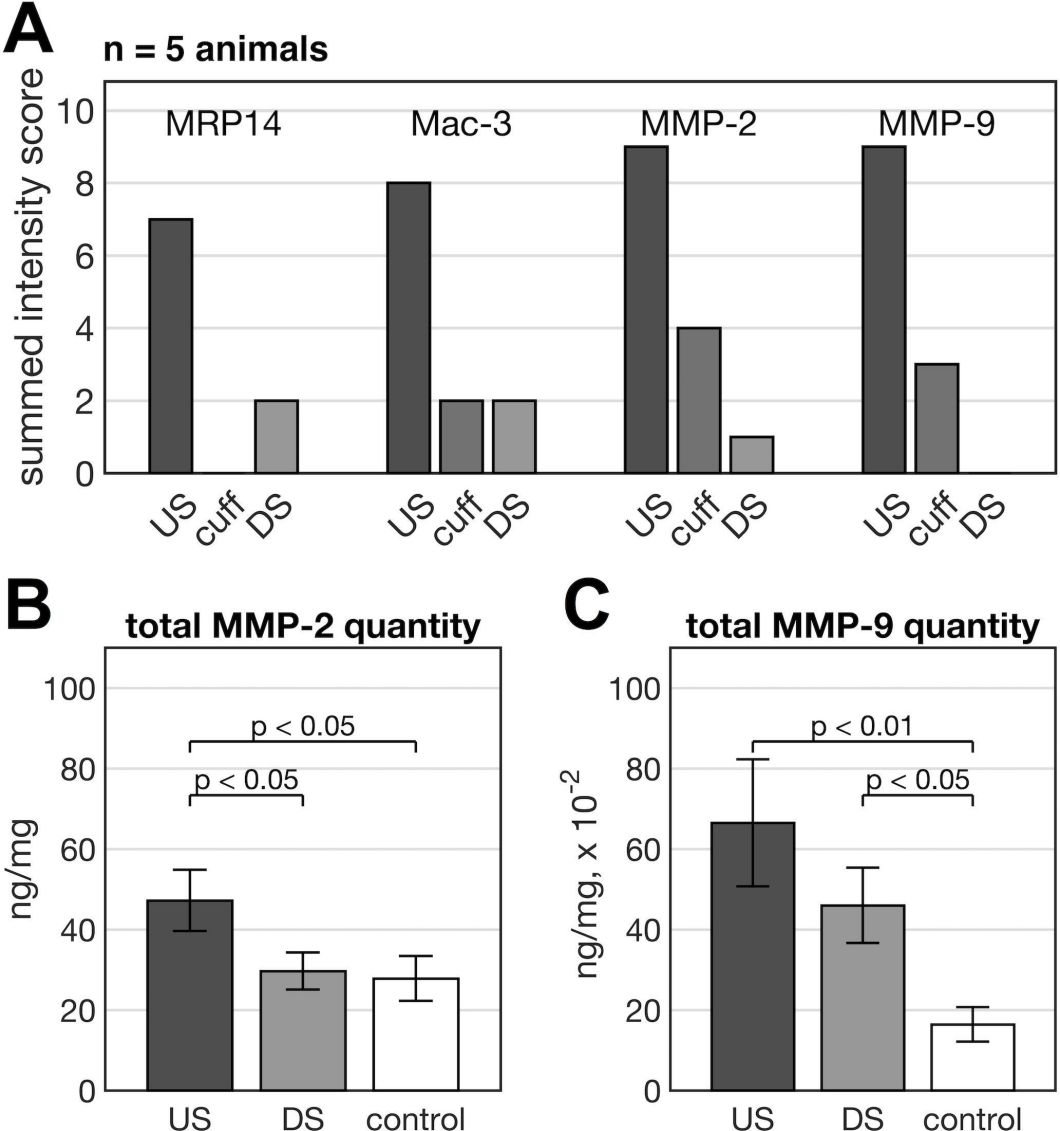
Histological and ELISA analysis of carotid arteries. Semi-quantitative intensity scores of the MMP-2, MMP-9, MRP14 and Mac-3 staining (n = 5) displayed as summed group score for DS, US and inside cuff regions (A). The summed group score is calculated by adding together the consensus reading results of all 5 animals, visualizing the results with respect to the performed staining and region (US, cuff or DS). Total MMP-2 (B) and MMP-9 (C) concentration normalised to protein level of right CCA US and DS region (n = 13) and left CCA as control (n = 14).

In US plaques, which exhibited a strong MMP signal in FRI imaging, the MMP-2 and -9 staining revealed strong MMP positivity together with a pronounced macrophage infiltration and activity (MRP14). This indicates unstable, highly inflamed plaque phenotypes. By contrast, DS plaques were smaller and exhibited a low MMP positivity and little to no macrophage infiltration and activity (“stable phenotype) nicely correlating to a significantly lower MMP imaging signal. Histology (n = 5 animals) was additionally semi-quantitatively analysed by expert readers supporting the characteristic features of cuff-induced plaques (Fig 5A).

### MMP concentrations measured by ELISAs

Since the differences in plaque size between US and DS plaques could (in part) explain the measured differences in tracer uptake (partial volume and unspecific tracer uptake) we finally aimed at quantitatively assessing the concentration of MMP-2 and MMP-9 in explanted carotid arteries, both in the US and DS plaques of the left CCA and the control right CCA. In accordance with the MMP signals measured by FRI, the MMP-2 concentration in the US plaque (47.2±7.6 ng/mg) was significantly higher than in the DS plaque (29.6±4.6 ng/mg; p<0.05) and the control left CCA (27.8±5.5 ng/mg; p<0.05) (Fig 5B). However, the MMP-2 concentration was similar between the DS plaque and the control. For MMP-9, the concentration was higher in the US and DS plaque as compared to the control left CCA (0.66±0.17 ng/mg vs. 0.44±0.10 ng/mg vs. 0.15±0.05 ng/mg; US vs. control p<0.01; DS vs. control p<0.05; Fig 5C), however, a statistical significance could not be established between US and DS plaques.

## Discussion

Here we report on the imaging of MMP activity using a fluorescent, MMP-directed small molecule tracer in cuff-bearing ApoE^-/-^ mice. The study’s aim was to evaluate MMP activity as an imaging biomarker to discriminate between stable and unstable or vulnerable plaque phenotypes. A plaque characterisation by means of molecular imaging is urgently needed to detect rupture prone atherosclerosis, which causes life threatening events. In this experimental model of atherosclerosis, we successfully prove and visualize overexpression of MMP-2 and MMP-9 in unstable upstream low oscillatory shear stress plaques as compared to the high laminar shear stress-induced downstream plaques and control carotids.

The tapered cuff model in ApoE^-/-^ mice nicely resembles features of human carotid stenosis, including reproducible changes in endothelial shear stress resulting in distinct flow patterns and plaque phenotypes–low laminar shear stress inducing unstable and vulnerable atherosclerotic plaques upstream, high laminar shear stress inside the cuff and oscillatory shear stress downstream promoting stable plaques phenotypes [11], [6]. Upstream of the tapered cuff, plaque formations in the CCA of ApoE^-/-^ mice on high-cholesterol diet show characteristic inflammatory macrophage activity [4], micro calcifications and zinc deposits in the intimal layer, which can also be found in human rupture prone plaques [12]. However, despite the many characteristics of rupture-prone plaques displayed by upstream lesions in the cuff model of ApoE^-/-^ mice, these plaques do not rupture. Thereby the model fails to emulate the full human pathogenesis [6]. In a first attempt of inflammation imaging in shear stress-induced atherosclerotic plaques, we have recently studied ApoE^-/-^ cuff mice 4, 6 and 8 weeks following cuff implantation by [^18^F]FDG-PET-CT [5]. This approach was based on successful examples of imaging macrophages in human atherosclerotic plaques by [^18^F]FDG-PET [13] [14]. We found a clearly enhanced [^18^F]FDG uptake correlating with a higher macrophage content in US plaques as compared to DS plaques, which increased from a 4 to an 8 week time point. However, [^18^F]FDG-PET is lacking specificity for inflammation or inflammatory cells by targeting a rather unspecific metabolic process (glucose uptake and phosphorylation), which especially in the case of post-surgical tissues or brown fat surrounding the cuff, could lead to relevant and unspecific background signals [15]. Furthermore, [^18^F]FDG is well correlated with the density of macrophages, whereas it might not reflect macrophage activity most probably more relevant for plaque instability. In addition, [^18^F]FDG uptake might also be enhanced in hypoxic atherosclerotic plaques [7].

Due to the lack of satisfactory imaging techniques, molecular targets for imaging the degree of plaque inflammation, which is associated with rupture have been proposed [16]. Amongst others, hypoxia markers or VCAMs seem to be promising targets [17] [18]. However, MMPs are particularly attractive since they are involved in a multitude of molecular mechanisms directly promoting plaque instability, including matrix degradation of the fibrous cap [19] [20] [21] [22]. For human carotid endatherectomy samples, it has been shown that MMPs are overexpressed and activated in rather vulnerable and symptomatic plaque states [2] [3]. Interestingly, MMP activity was associated with a thin fibrous cap and a large lipid core phenotype of carotid plaques making MMP activity a highly promising molecular target for molecular imaging [2].

In a first attempt Jager et al. have recently tested the feasibility of imaging MMP activity in human carotid atherosclerosis by *in vitro* incubation of fresh endatherectomy samples with an activatable fluorescent MMP probe sensitive for MMP-2 and MMP-9. They found significant tracer activation in intraluminal, but also extraluminal areas of carotid plaques, which correlated with MMP-9 overexpression and presence of CD-68 positive macrophages [23]. However, using human samples, Jager et al. could not study MMPs’ imaging specificity for rupture prone plaques. This limitation would be conquered by the simultaneous imaging of MMP activity in stable and unstable plaques, which is done by our study.

Yet translating activatable fluorescent peptidic probes such as MMPSense^TM^ 680 used in Jager’s study into clinical applications is still hampered by scattering of fluorescence signals in deep tissues such as the carotid arteries and regulatory pharmaceutical issues. The latter could be circumvented by using small molecule MMP inhibitors as probes that bind to active MMPs [24] [25]. Usually, also in this study, these molecules show a broad-spectrum activity including gelatinases and collagenases and can be labelled by various moieties for different imaging modalities (fluorescent dyes, isotopes etc.). We therefore aimed to test the feasibility of imaging MMP activity in carotid plaques by a barbiturate-based fluorescent tracer (Cy5.5-AF443) developed by our group and applied in different pathologies such as tumours and vasculitis models [8] [26] [27].

For this purpose, we employed the carotid cuff model in ApoE^-/-^ mice which reproducibly results in shear stress-dependent distinct plaque phenotypes and a reliable control tissue given the unaffected left carotid artery. In this model, immunohistochemistry of cuff-related plaques has recently shown an increased MMP-9 concentration in the US plaque, which could be suppressed by pharmaceutical intervention with a angiotensin-converting enzyme 2 inhibitor [28]. For analysing differences between US and DS plaques with respect to the presence of MMP and to test the feasibility of MMP imaging to characterize these plaques *in vivo*, we employed cuff-bearing ApoE-/- mice 8 weeks after cuff implantation. This time point has been shown to feature the highest atherosclerotic inflammation [5]. Upon injection of our non-peptidic MMP tracer (Cy5.5-AF443) in this carotid stenosis model, we find a significantly enhanced fluorescence signal in the US plaque as compared to its DS counterpart *in situ* suggesting that imaging of MMP activity could distinguish unstable ‘MMP-rich’ plaques from stable phenotypes. Interestingly, the US and DS P/B ratios are even higher *ex vivo* compared to the *in situ* measurement. This might be explained partly by unspecific uptake in surrounding tissues (background activity), which is removed for the *ex vivo* measurement and by shrinking artefacts *ex vivo* (partial volume effects). Correlative immunohistological stainings show a match of the presence of MMP-2, MMP-9 and activated macrophages (Mac-3 and MRP14) and the Cy5.5-AF443 fluorescence signal, with higher MMP-2 and MMP-9 expression in the US region. The US region does also feature a higher amount of macrophages consistent with a larger plaque size. Moreover, plaque size and Cy5.5-AF443 fluorescence P/B ratio showed a good correlation. While this indicates that unstable and larger plaques are associated with MMP activity, partial volume effects could also partly contribute to this finding. To assess the contribution of potential unspecific tracer uptake in plaque formations, we used the non-targeted identical fluorophore Cy5.5 as a control dye. This control dye revealed no significant difference in uptake comparing US and DS plaques, further corroborating the specificity of the Cy5.5-AF443 tracer.

To further validate the specificity of the MMP imaging signal we determined absolute MMP-2 and MMP-9 concentration by ELISA, corrected for protein content. Thereby increased MMP levels were confirmed in US plaques as compared to DS plaques, which further supported the validity of the cuff model for mimicking features of the human scenario. This finding also confirms the presented histological results. However, our analyses revealed that, although both MMP subtypes are increased compared to the DS plaque, the absolute MMP-2 concentration in the US region is approximately 100 times higher compared to the MMP-9 concentration. This indicates that in the cuff model the Cy5.5-AF443 fluorescence signal is likely to stem primarily from MMP-2. There was no statistically significant difference comparing MMP-9 activity of US and DS plaques, yet the US activity is greater on average. This is confirmed by the histological analysis that shows more MMP-9 positive staining in the US position.

The perivascular inflammation induced by the surgical implantation of the tapered cuff may distort the findings, as it could introduce non-atherosclerosis related background signals for imaging, but also promote the atherosclerotic development. However, this would affect both the US and DS regions to an equal extent. More importantly, perivascular inflammation in atherosclerotic lesions can also be observed in other atherosclerosis models [29]. Though the unstable and stable plaques in this murine cuff model feature a well described human like phenotype, murine vessels cannot fully mirror the human vasculature due to a disproportion of plaque to vessel size and missing rupture events.

In this feasibility study, we have employed a fluorescence-based imaging approach, which allows a correlation to histology and quantitative tissue analysis, directly proofing that MMP imaging can discriminate plaque phenotypes. The fluorescent imaging approach enables unparalleled spatial image resolution of the vascular inflammation *in vivo*. Yet, even in mice, fluorescence-based imaging approaches are not suited for cardiovascular serial imaging, since emission signals from deep structures such as the carotid arteries are heavily scattered especially in black mice such as the ApoE^-/-^ model. There are several strategies dedicated to optical imaging of atherosclerosis *in vivo* [30]. These approaches usually utilize Fluorescence Molecular Tomography (FMT) or photoacoustic imaging. The possibility of *in vivo* tracer quantification is of great importance, not only to allow sequential imaging, but also for therapy response studies. Image modalities like FMT and photoacoustics need a rigorous validation using *in situ* FRI measurements, as they are susceptible to optical artefacts. Such artefacts are severely introduced by the here employed plastic cuff, which on the one hand causes shear stress induced atherosclerotic lesions, but on the other hand also provokes extinction and scattering artefacts. Thereby, the cuff model hampers a sound quantification when using FMT or photoacoustics. Thus, in this feasibility study, we relied on *in situ* and *ex vivo* FRI imaging, ensuring unbiased measurements.

Still, the use of a photoprobe is not far away from a clinical application, as invasive catheter based methods are already in use, enabling the optical assessment of the vascular interior [31] [32] [33]. However, exchanging the fluorescent dye label by radioactive isotopes should allow longitudinal non-invasive PET and SPECT (Single-photon emission computed tomography) imaging in mice and approaching clinical translation, as we have recently shown for assessing MMP activity in Multiple Sclerosis patients [34].

## Conclusion

Imaging of MMP activity using a Cy5.5-labeled broad spectrum MMP inhibitor is able to distinguish low shear stress induced vulnerable plaques from stable plaque phenotypes in the carotid artery of cuff-bearing ApoE^-/-^ mice. In this model MMP-2 concentration is higher in vulnerable plaques as compared to MMP-9.

## Supporting information

S1 Fig Control dye uptake in plaque formations. Panel A depicts the situs of 4 mice prior to *in situ* imaging together with a schematic drawing. Dashed lines separate the three vessel sections, upstream (US), cuff and downstream (DS) region. In all mice a pronounced US plaque formation is visible while the DS plaque development is less severe. However, FRI in the same animals as in A injected with the non-targeted Cy5.5-glycin dye alone shows no difference in dye uptake comparing US and DS plaques (B). In contrast, representative *in situ* measurements of 4 additional mice injected and scanned with the MMP-targeted Cy5.5-AF443 tracer are shown as well show an increased MMP tracer accumulation in the US plaque compared to DS further proofing MMP-specific binding of Cy5.5-AF443. Plaque to background ratios are used as image units for all presented fluorescent images.
(TIFF)

S2 Fig Detailed histological staining. Magnification from an upstream plaque formation. The magnified area is depicted by dashed rectangle in the HE image (Scale bar = 150 𝜇m). The arrowheads point out colocalization of antibody binding and nuclei, indicating specific staining.
(TIFF)

S3 Fig Additional histological staining. Adjacent slices of an US plaque formation are stained for Mac3/MRP14 (A) and MMP-2 (B) respectively. DS (C) and US plaque (D) from one mouse stained for Mac3 and MRP14.
(TIFF)

S1 File Relevant data. (XLSX)
