# Supplementary figures and images for: Molecular imaging of MMP activity discriminates unstable from stable plaque phenotypes in shear-stress induced murine atherosclerosis
Source: PLoS One. 2018 Oct 10;13(10):e0204305. doi: 10.1371/journal.pone.0204305 (PMC6179381; doi:10.1371/journal.pone.0204305)

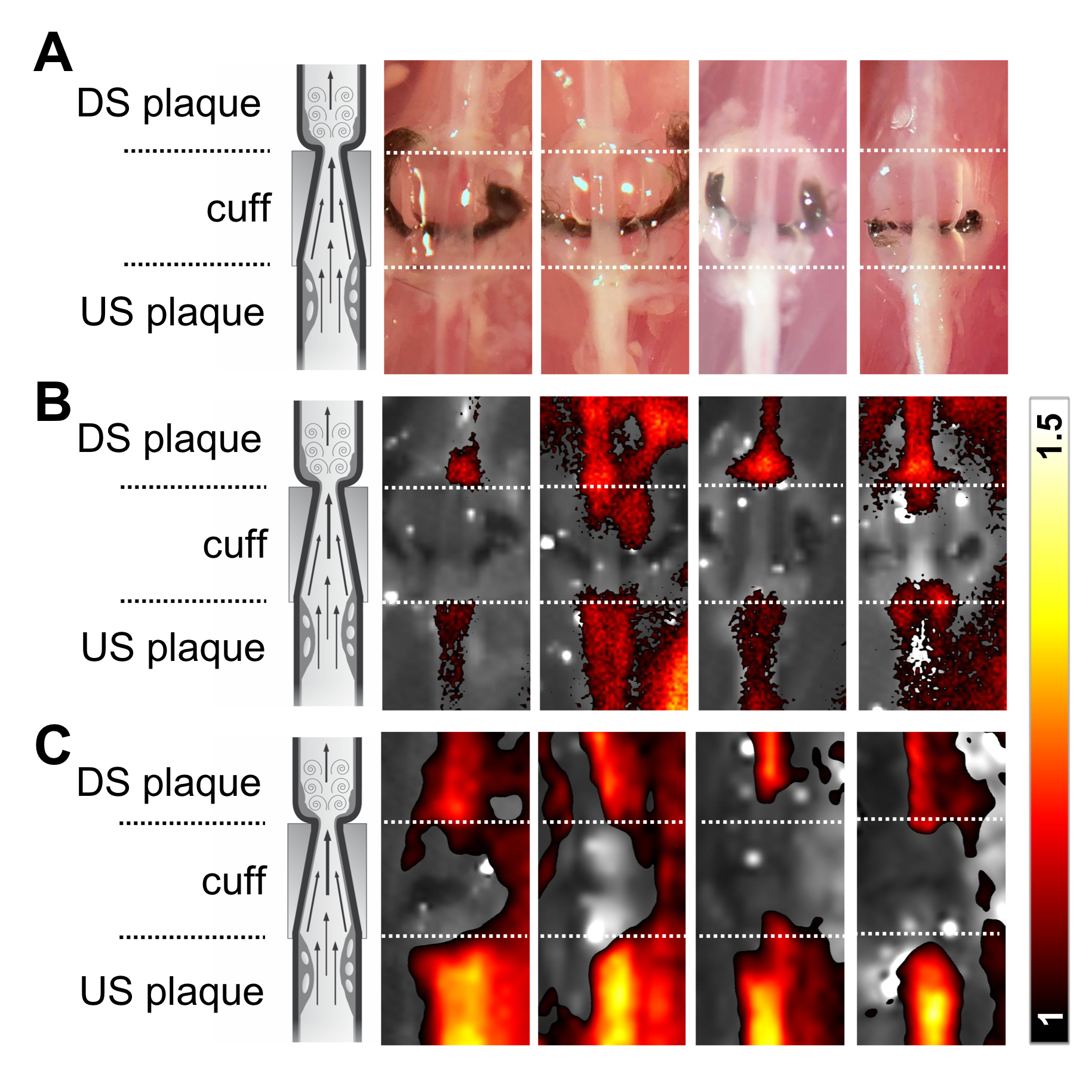

Supplement: S1 Fig — Panel A depicts the situs of 4 mice prior to in situ imaging together with a schematic drawing. Dashed lines separate the three vessel sections, upstream (US), cuff and downstream (DS) region. In all mice a pronounced US plaque formation is visible while the DS plaque development is less severe. However, FRI in the same animals as in A injected with the non-targeted Cy5.5-glycin dye alone shows no difference in dye uptake comparing US and DS plaques (B). In contrast, representative in situ measurements of 4 additional mice injected and scanned with the MMP-targeted Cy5.5-AF443 tracer are shown as well show an increased MMP tracer accumulation in the US plaque compared to DS further proofing MMP-specific binding of Cy5.5-AF443. Plaque to background ratios are used as image units for all presented fluorescent images. (TIFF) [file pone.0204305.s001.tiff]

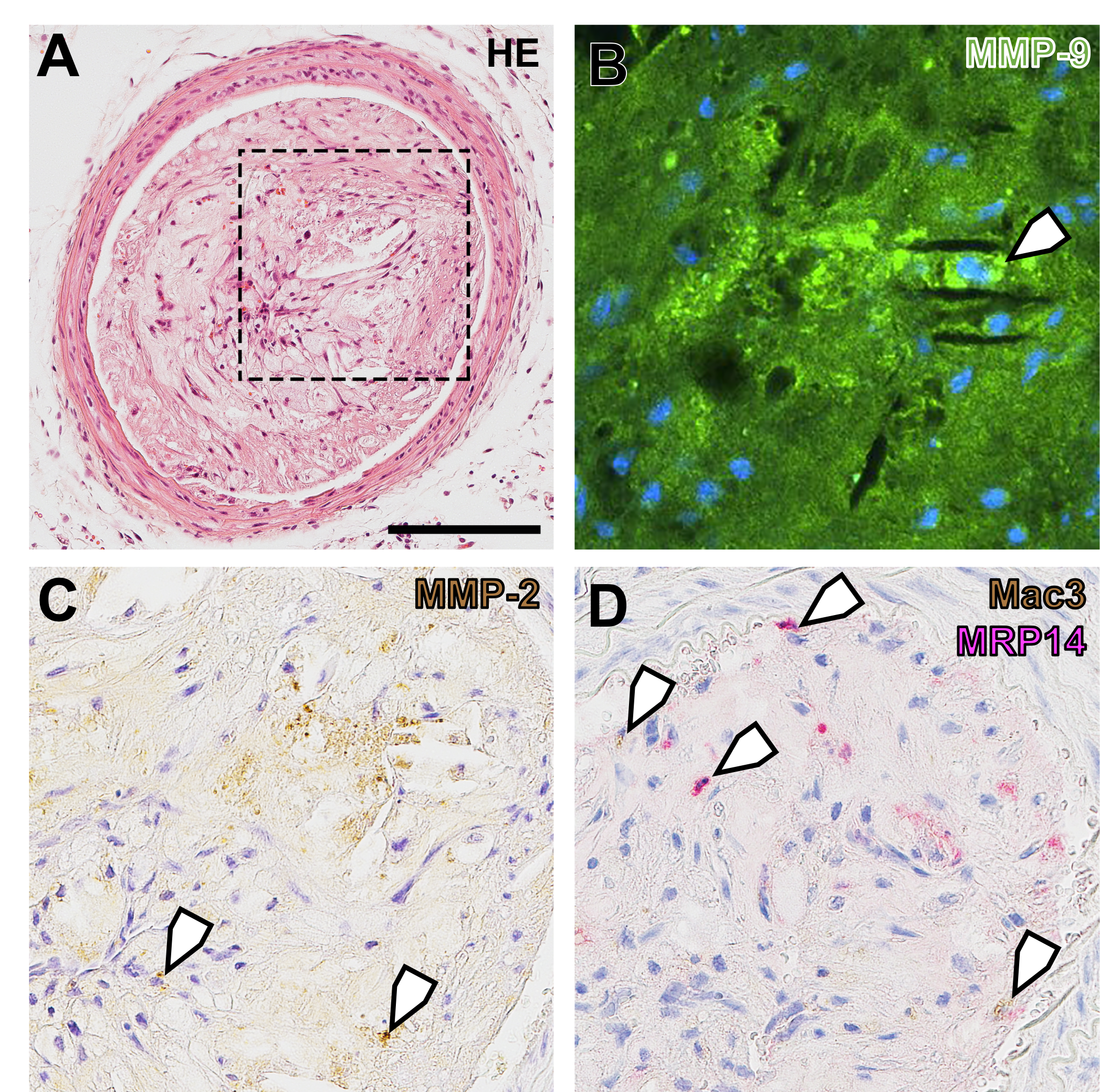

Supplement: S2 Fig — Magnification from an upstream plaque formation. The magnified area is depicted by dashed rectangle in the HE image (Scale bar = 150 𝜇m). The arrowheads point out colocalization of antibody binding and nuclei, indicating specific staining. (TIFF) [file pone.0204305.s002.tiff]

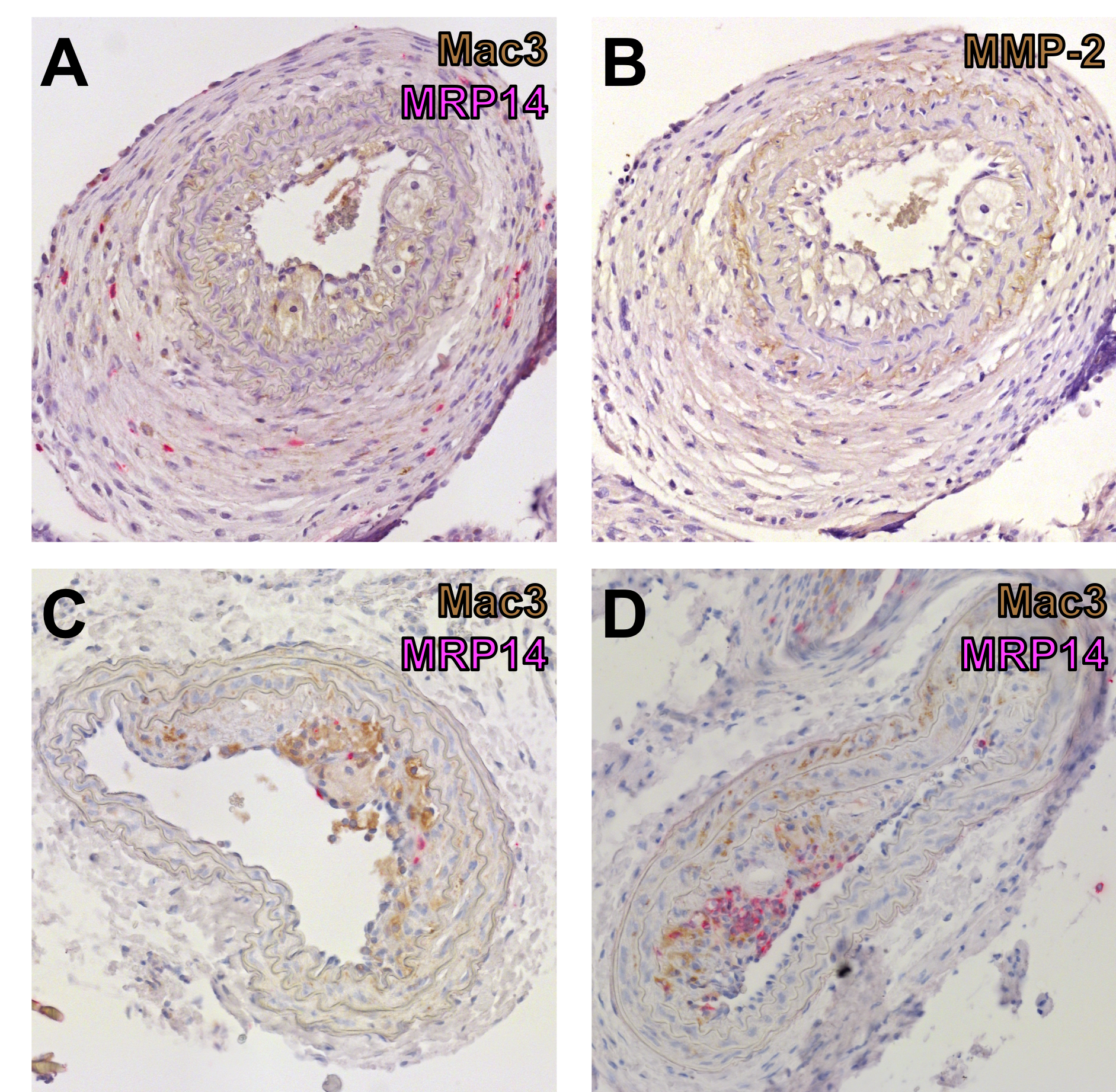

Supplement: S3 Fig — Adjacent slices of an US plaque formation are stained for Mac3/MRP14 (A) and MMP-2 (B) respectively. DS (C) and US plaque (D) from one mouse stained for Mac3 and MRP14. (TIFF) [file pone.0204305.s003.tiff]
